# Improved First-Principles Calculation of the Third Virial Coefficient of Helium

**DOI:** 10.6028/jres.116.016

**Published:** 2011-08-01

**Authors:** Giovanni Garberoglio, Michael R. Moldover, Allan H. Harvey

**Affiliations:** Interdisciplinary Laboratory for Computational Science (LISC), FBK-CMM and University of Trento, Via Sommarive 18, I-38123 Povo, Italy; Temperature, Pressure, and Flow Metrology Division, National Institute of Standards and Technology, Gaithersburg, MD 20899; Thermophysical Properties Division, National Institute of Standards and Technology, Boulder, CO 80305

**Keywords:** coustic virial coefficients, calibration, density, helium, metrology, thermodynamic properties, virial coefficients

## Abstract

We employ state-of-the-art pair and three-body potentials with path-integral Monte Carlo (PIMC) methods to calculate the third density virial coefficient *C*(*T*) for helium. The uncertainties are much smaller than those of the best experimental results, and approximately one-fourth the uncertainty of our previous work. We have extended our results in temperature down to 2.6 K, incorporating the effect of spin statistics that become important below approximately 7 K. Results are given for both the ^3^He and ^4^He isotopes. We have also performed PIMC calculations of the third acoustic virial coefficient *γ*_a_; our calculated values compare well with the limited experimental data available. A correlating equation for *C*(*T*) of ^4^He is presented; differentiation of this equation provides a reliable and simpler way of calculating *γ*_a_.

## 1. Introduction

Accurate knowledge of the thermophysical properties of helium is desirable for many applications in metrology [1–3]. At low and moderate densities, thermodynamic properties are well described by the virial expansion, which gives a rigorous series of corrections to ideal-gas behavior:

(1) $$\frac{p}{\rho RT}=1+B\left(T\right)\rho +C\left(T\right)\rho^{2}+\ldots$$

In Eq. (1)*p* is the pressure, *ρ* the molar density, *R* the molar gas constant, and *T* the absolute temperature. The second virial coefficient *B*(*T*) depends only on interactions between pairs of molecules, while the third virial coefficient *C*(*T*) depends on interactions among three molecules.

In 2009, two of us reported [4] path-integral Monte Carlo (PIMC) calculations of the third virial coefficient *C*(*T*) for ^4^He at temperatures from 24.5661 K (corresponding to the triple point of neon) to 10 000 K. The values of *C*(*T*) reported in Ref. [4] were based on a representation of the pair potential [5] and a three-body potential [6] that were state-of-the-art (or nearly so) at the time the work was performed.

The uncertainties obtained in Ref. [4] were smaller than those of the best experimental results by approximately an order of magnitude, with the majority of the uncertainty coming from the three-body potential. Further improvement is desirable—for example, in a proposed pressure standard based on capacitance measurement at 273.16 K, the uncertainty in *C* is still the largest contributor to the uncertainty budget between approximately 8 MPa and 20 MPa [7]. It is also desirable to extend the results to lower temperatures, where helium plays an important role in temperature metrology.

Recently, a state-of-the-art pair potential for helium has been developed [8, 9]. The new potential incorporates not only extremely accurate results for the potential energy in the Born-Oppenheimer (BO) approximation [10], but also accurate calculations for the most important post-BO effects (adiabatic, relativistic, and quantum electrodynamics). The claimed uncertainty of the new pair potential is at least one order of magnitude smaller than that of the potential we used in Ref. [4]. This pair potential has been used to obtain highly accurate values for the second virial coefficient *B*(*T*) and for the low-density limits of the viscosity and thermal conductivity [9]. In addition, a new three-body potential has been developed at the full-configuration-interaction (FCI) level [11], reducing the uncertainty of the three-body potential by approximately a factor of five compared to that used in Ref. [4].

In this work, we take advantage of the availability of these better potentials, and of increased computing capabilities and algorithmic improvements, to recalculate *C*(*T*) with lower uncertainty than could be obtained in Ref. [4] and to extend our calculations to lower temperatures. We also extend our work to the third acoustic virial coefficient, and give some results for the ^3^He isotope. In this paper, we will focus on aspects that differ from Ref. [4], such as the calculation of acoustic virials and the low-temperature results. The reader is referred to Ref. [4] for further background, a literature review, and details of the uncertainty analysis. Some additional details of the PIMC calculations, especially at low temperatures where spin statistics become important, are discussed in Ref. [12].

## 2. Intermolecular Potentials

For the pair potential, we use the potential first presented by Przybytek et al. [8] and described in more detail by Cencek et al. [9]. A function for the uncertainty of this potential is given in the Supplemental Material for Ref. [8], so that upper- and lower-bound potentials can be obtained by adding or subtracting the uncertainty function from the recommended pair potential. While Przybytek et al. do not attach a rigorous statistical interpretation to their “uncertainty,” we believe that it is reasonable to treat it as an expanded uncertainty with coverage factor *k* = 2, which corresponds to a 95 % confidence limit. For calculations with ^3^He, a small adjustment (negligible in the context of this work) was made to scale the adiabatic correction to the pair potential [9] to account for the different mass.

For the nonadditive three-body potential of helium, we use the FCI potential of Cencek et al. [11]. This potential is stated to have a relative uncertainty of 2 %, which again we interpret as an expanded uncertainty at the *k* = 2 level. For our uncertainty analysis, we construct perturbed three-body potentials FCI− (obtained by multiplying the corresponding potential by 1.02 where it is negative and by 0.98 where it is positive) and FCI+ (multiplying by 0.98 where it is negative and by 1.02 where it is positive).

## 3. Calculation Methods

### 3.1 Third Density Virial Coefficient

It has been shown in Refs. [4] and [12] that the second virial coefficient *B*(*T*) and the third virial coefficient *C*(*T*) for a quantum system obeying Boltzmann statistics can be written as

(2) $$B\left(T\right)=-2\pi N_{A}\int r^{2}dr\left[e^{-\beta {\bar{U}}_{2}\left(r\right)}-1\right]$$

(3) $$C\left(T\right)=4B^{2}\left(T\right)-\frac{N_{A}^{2}}{3}\int dr_{1}dr_{2}\left[e^{-\beta {\bar{V}}_{3}\left(r_{1},r_{2}\right)}-e^{-\beta {\bar{U}}_{2}\left(\left|r_{1}-r_{2}\right|\right)}-e^{-\beta {\bar{U}}_{2}\left(\left|r_{1}\right|\right)}-e^{-\beta {\bar{U}}_{2}\left(\left|r_{2}\right|\right)}+2\right],$$

where *N*_A_ is Avogadro’s constant, *β* = 1/*k*_B_*T*, and the effective two-body and three-body potentials ${\bar{U}}_{2}\left(r\right)$ and ${\bar{V}}_{3}\left(r_{1},r_{2}\right)$ are given by the following path-integral expressions:

(4) $$\begin{array}{l} \exp\left(-\beta {\bar{U}}_{2}(r)\right)=\\ \;\oint Dx_{1}Dx_{2}\exp\left[-\frac{1}{\hbar }\int_{0}^{\beta \hbar }\frac{m}{2}\left({\left|\frac{dx_{1}(\tau )}{d\tau }\right|}^{2}+{\left|\frac{dx_{2}(\tau )}{d\tau }\right|}^{2}\right)+U_{2}\left(\left|r+x_{1}(\tau )-x_{2}(\tau )\right|\right)d\tau \right] \end{array}$$

(5) $$\exp\left[-\beta {\bar{V}}_{3}(r_{1},r_{2})\right]=\oint Dx_{1}Dx_{2}Dx_{3}\exp\left[-\frac{1}{\hbar }\int_{0}^{\beta \hbar }\frac{m}{2}\left({\left|\frac{dx_{1}(\tau )}{d\tau }\right|}^{2}+{\left|\frac{dx_{2}(\tau )}{d\tau }\right|}^{2}+{\left|\frac{dx_{3}(\tau )}{d\tau }\right|}^{2}\right)+V_{3}\left(r_{1}+x_{1}(\tau ),r_{2}+x_{2}(\tau ),x_{3}(\tau )\right)d\tau \right],$$

where *U*_2_(*r*) and *V*_3_(***r***_1_,***r***_2_,***r***_3_) are the two-body and three-body potential energies, respectively, and *m* is the particle mass. In Eq. (5), the position of particle 3 in the *τ* = 0 imaginary-time slice has been fixed at the origin of the coordinate system, due to the translational invariance of the integrand. The variables ***r***_1_ and ***r***_2_ reduce to the position of particles 1 and 2, respectively, in the classical limit, that is, when *T* is so high that the paths *x_k_*(*τ*) (*k* = 1, 2, 3) contributing most to the path integrals shrink to a point. A similar procedure was followed for Eq. (4), where we used the translational invariance of the integrand to fix the position of particle 2 in the *τ* = 0 imaginary-time slice at the origin. The variable ***r*** in Eq. (4) denotes the position of particle 1 in the classical limit.

The three-body potential energy is given by

(6) $$V_{3}(r_{1},r_{2},r_{3})=U_{3}(r_{1},r_{2},r_{3})+U_{2}(\left|\left(r_{1}-r_{2}\right)\right|)+U_{2}(\left|\left(r_{2}-r_{3}\right)\right|)+U_{2}(\left|\left(r_{1}-r_{3}\right)\right|),$$

where *U*_3_(***r***_1_, ***r***_2_, ***r***_3_) denotes the non-additive part of the three-body potential energy and *U*_2_(r) is the pair potential. In Eqs. (4) and (5), the path integrals are performed over all closed paths that have the origin as endpoints, that is, paths ***x***(*τ*) fulfilling the conditions ***x***(0) = ***x***(*βħ*) = **0**. The path integrals are normalized in such a way that their discretized form reads [12]

(7) $$\oint Dx\exp\left[-\frac{1}{\hbar }\int_{0}^{\beta \hbar }\frac{m}{2}{\left|\frac{dx_{1}(\tau )}{d\tau }\right|}^{2}d\tau \right]\equiv \underset{P\to \infty }{\lim}\int \prod_{i=2}^{P}dx^{\left(i\right)}\Lambda^{3}{\left(\frac{P^{3/2}}{\Lambda^{3}}\right)}^{P}\exp\left[-\frac{\pi P}{\Lambda^{2}}\sum_{i=1}^{P}{\left|x^{\left(i+1\right)}-x^{\left(i\right)}\right|}^{2}\right]=1,$$

where ***x***^(^*^P^*^+1)^ = ***x***^(1)^ = **0** and $\Lambda =h/\sqrt{2\pi mk_{B}T}$ is the de Broglie wavelength of a particle with mass *m*. The integrand of the middle expression in Eq. (7) can be interpreted as the probability of having a ring polymer with *P* beads in the positions given by the coordinates ***x***^(1)^,…,***x***^(P)^ [13]. In the following subsection, this quantity will be denoted as *F*_ring_.

At low temperatures where Boltzmann statistics is no longer a good approximation, the above equations must be extended to incorporate Bose-Einstein or Fermi-Dirac statistics. The details of this extension are given in Ref. [12], where it was shown that the incorporation of spin statistics is necessary for both isotopes of helium at temperatures below approximately 7 K.

The number of beads *P* was chosen as a function of temperature according to *P* = 7 + (1200 K)/*T* for ^4^He and *P* = 7 + (1800 K)/*T* for ^3^He, with the resulting *P* rounded to the nearest integer. Preliminary calculations showed that these choices for *P* provide converged results (well within the statistical uncertainty of the calculation) for *C*(*T*) throughout the range of temperatures spanned by the present work. The ring polymers were generated as described in Ref. [4]. As in Ref. [4], we used the VEGAS algorithm [14] for the numerical integration of Eq. (7). We averaged 256 independent calculations, each with 10^6^ integration points, in order to obtain the final result and its statistical uncertainty. For low temperatures where spin statistics are significant, the additional non-Boltzmann terms required were calculated as described in Ref. [12] with 128 independent integrations.

### 3.2 Third Acoustic Virial Coefficient

The square of the speed of sound in a gas as a function of pressure on isotherms has the low-density expansion:

(8) $$u^{2}=\frac{\gamma_{0}RT}{M}\left(1+\frac{\beta_{a}p}{RT}+\frac{\gamma_{a}p^{2}}{RT}+\frac{\delta_{a}p^{3}}{RT}+\cdots \right).$$

Here, *u* is the speed of sound; *β*_a_, *γ*_a_, and *δ*_a_ are the temperature-dependent second, third, and fourth acoustic virial coefficients; *M* is the molar mass; and *γ*_0_ ≡ *C_p_*/*C_v_* is the ratio of the constant-pressure heat capacity to the constant-volume heat capacity in the ideal-gas state, which is exactly 5/3 for a monatomic gas. [The ideal-gas heat-capacity ratio *γ*_0_ should not be confused with the acoustic virial coefficient *γ*_a_.] Insofar as *γ*_a_ is a second-order correction for non-ideality, it is analogous to the third density virial coefficient *C*. We choose to discuss *RTγ*_a_ instead of *γ*_a_ because *RTγ*_a_ has both the units and the order of magnitude of the more familiar third density virial coefficient *C*. Exact thermodynamic relations connect *γ*_a_ to the density virial coefficients *B* and *C* and their first two temperature derivatives [15]. These relations are:

(9) $$RT\gamma_{a}=L-\beta_{a}B,$$

where *β*_a_ is related to the second density virial coefficient *B*(*T*) and its temperature derivatives by

(10) $$\beta_{a}(T)=2B+2\left(\gamma_{0}-1\right)T\frac{dB}{dT}+\frac{{\left(\gamma_{0}-1\right)}^{2}}{\gamma_{0}}T^{2}\frac{d^{2}B}{dT^{2}},$$

and

(11) $$\gamma_{0}L\left(T\right)=\left(\gamma_{0}-1\right)Q^{2}+\left(2\gamma_{0}+1\right)C+\left(\gamma_{0}^{2}-1\right)T\frac{dC}{dT}+\frac{{\left(\gamma_{0}-1\right)}^{2}}{2}T^{2}\frac{d^{2}C}{dT^{2}},$$

(12) $$Q\left(T\right)=B+\left(2\gamma_{0}-1\right)T\frac{dB}{dT}+\left(\gamma_{0}-1\right)T^{2}\frac{d^{2}B}{dT^{2}}.$$

For PIMC calculation of the acoustic virial coefficients, it is necessary to derive path-integral expressions for the temperature derivatives of the density virial coefficients. For this purpose, we use the derivatives of Eqs. (2) and (3) with respect to *β* = 1/*k*_B_*T*, together with the identities

(13) $$\frac{d}{dT}=-\beta^{2}\frac{d}{d\beta }$$

(14) $$\frac{d^{2}}{dT^{2}}=\beta^{3}\left(2\frac{d}{d\beta }+\beta \frac{d^{2}}{d\beta^{2}}\right).$$

In Eqs. (2) and (3), the second and third density virial coefficients are given as a sum of terms that involve the integral of products of ring-polymer probability distributions with Boltzmann factors of the interaction potential averaged along the path. Making use of the fact that $\frac{d\Lambda }{d\beta }=\frac{\Lambda }{2\beta }$, the derivatives of the ring-polymer probability distribution can be written as

(15) $$\frac{dF_{\text{ring}}}{d\beta }=\frac{1}{\beta }\left[\frac{\pi P}{\Lambda^{2}}\sum_{i=1}^{P}{\left|x^{\left(i+1\right)}-x^{\left(i\right)}\right|}^{2}-\frac{3}{2}\left(P-1\right)\right]F_{\text{ring}}\equiv \frac{a}{\beta }F_{\text{ring}},$$

where the last equality defines the quantity *a*. The Boltzmann factors of the potential along the paths quite generally have the form

(16) $$W\equiv e^{-\beta \bar{U}(r)}=\exp\left[\frac{-\beta }{P}\sum_{i=1}^{P}U\left(\left|r+x^{\left(i\right)}\right|\right)\right],$$

whose *β* derivative is given by

(17) $$\frac{{\text{de}}^{-\beta \bar{U}(r)}}{d\beta }=-\frac{1}{P}\sum_{i=1}^{P}U\left(\left|r+x^{\left(i\right)}\right|\right)e^{-\beta \bar{U}(r)}\equiv \frac{b}{\beta }W,$$

where the last equality defines the quantity *b*.

Path-integral expressions for the temperature derivatives of *B*(*T*) and *C*(*T*) can then be derived with the use of Eqs. (15) and (17). For example, the first temperature derivative of *B*(*T*) can be written as

(18) $$\begin{array}{l} \frac{dB}{dT}=-2\pi N_{A}\beta \int r^{2}dr\prod_{i=2}^{P}dx_{1}^{\left(i\right)}dx_{2}^{\left(i\right)}\\ \;F_{\text{ring}}(1)F_{\text{ring}}(2)\left[\left(a_{1}+a_{2}\right)\left(W-1\right)+bW\right], \end{array}$$

where *F*_ring_(1) is the probability distribution for the configurations of the first ring polymer, with a corresponding definition for the second, and *a*_1_ and *a*_2_ are the quantity *a* defined in Eq. (15) for the first and second ring polymer, respectively.

In the case of *C*(*T*), Eq. (18) can be modified to calculate the temperature derivatives of the terms appearing in Eq. (3), taking into account the fact that the potential in the definition of *W* is actually a three-body potential and that three ring polymers must be considered. As a consequence, there is another distribution probability *F*_ring_(3) for the third particle, as well as an integration over these ring-polymer configurations (that is,an additional factor $\prod_{i=2}^{P}dx_{3}^{\left(i\right)}$ in the integration measure). Moreover, *a*_1_ + *a*_2_ must be replaced by *a*_1_ + *a*_2_ + *a*_3_, and the integral over d*r* in Eq. (18) becomes an integration over d***r***_1_d***r***_2_ when calculating d*C*/d*T*.

To calculate the second temperature derivatives of the virial coefficients, we use Eq. (14) together with the relations

(19) $$\frac{d^{2}F_{\text{ring}}}{d\beta^{2}}=\frac{F_{\text{ring}}}{\beta^{2}}\left[a^{2}-2a-\frac{3}{2}\left(P-1\right)\right]$$

(20) $$\frac{d^{2}W}{d\beta^{2}}=\frac{W}{\beta^{2}}b\left(b-1\right).$$

The final result for the second derivative of *B*(*T*) is

(21) $$\begin{array}{l} \frac{d^{2}B}{dT^{2}}=-2\pi N_{A}\beta^{2}\int r^{2}dr\prod_{i=2}^{P}dx_{1}^{\left(i\right)}dx_{2}^{\left(i\right)}F_{\text{ring}}(1)F_{\text{ring}}(2)\\ \;\left[\left({\left(a_{1}+a_{2}\right)}^{2}-3\left(P-1\right)\right)\left(W-1\right)+W\left(b^{2}+2\left(a_{1}+a_{2}\right)b+b\right)\right]. \end{array}$$

The second derivative of *C*(*T*) is given by a similar expression, after performing on Eq. (21) the same substitutions described above for the first derivative, together with replacing the term 3(*P* − 1) by 9(*P* − 1)/2.

The temperature derivatives of *C*(*T*) were used to calculate *L*(*T*) according to Eq. (11). The same PIMC methodology was used as described in Sec. 3.1; the values were obtained from 256 independent calculations with 10^6^ integration steps each.

## 4. Results

### 4.1 Third Virial Coefficients

Table 1 shows our calculated *C*(*T*) for ^4^He. In addition to all the temperatures given in Ref. [4], Table 1 includes lower temperatures (including some corresponding to fixed points on the ITS-90 temperature scale) and a few additional intermediate temperatures. Spin statistics significantly affect *C*(*T*) below about 7 K; this is discussed in detail in Ref. [12], where we describe the method of incorporating these effects and show the size of the various exchange contributions at low temperatures for both ^3^He and ^4^He.

The low-temperature values in Table 1 differ slightly from those given in Ref. [12]; we discovered a small error in our earlier implementation of the three-body potential and the values in Table 1 supersede those in Ref. [12]. These differences are smaller than the uncertainties of the calculated *C*(*T*), and the conclusions of Ref. [12] are not affected.

### 4.2 Third Acoustic Virial Coefficients

In Table 2, we show results for the third acoustic virial coefficient *γ*_a_. Because the quantity actually calculated by PIMC is *L* (see Sec. 3.2 and Eqs. (9) and (11)), and because of the variation of the magnitude of *γ*_a_ with temperature, we tabulate the quantity *RTγ*_a_ (and its expanded uncertainty as discussed below). Calculation of *RTγ*_a_ via Eq. (9) requires values of *B* and *β*_a_, which we obtain from the work of Cencek et al. [9] and which have such small uncertainties that they can be considered exact in the context of these calculations of *γ*_a_.

The uncertainty in the path-integral calculation of *γ*_a_ becomes quite large at low temperatures, which is why PIMC values at lower temperatures are not reported in Table 2. This is due to the statistical uncertainty of the Monte Carlo integration for *L*; the convergence behavior of this integration is much worse than that for *C*(*T*). The reason for this difference is not completely clear, but it may be due to the fact that the quantity *a* that is averaged in the calculation of d*C*/d*T* [see Eq. (15)] is the so-called thermodynamic estimator of the kinetic energy. This estimator is known to be characterized by a large variance, and therefore long computations are needed to evaluate its average value with small uncertainty [16]. Moreover, the second temperature derivative needed for calculation of the acoustic virial coefficients might add further statistical noise to the calculation.

It is also possible to calculate *γ*_a_ from a correlation of *C*(*T*), as given in Sec. 4.4, by differentiating the correlation to produce d*C*/d*T* and d^2^*C*/d*T*^2^ as required in Eq. (11). The quantities involving *B* can again be obtained from the work of Cencek et al. [9]. Values of *RTγ*_a_ calculated in this manner are also shown in Table 2. They are consistent with the values calculated directly by PIMC.

### 4.3 Uncertainty Analysis

The analysis of uncertainty in *C*(*T*) was similar but not identical to that described in Ref. [4]. The contributing factors are the uncertainty in the pair potential, the uncertainty in the three-body potential, and the uncertainty in the convergence of the PIMC calculation.

The standard uncertainty due to PIMC convergence was estimated as the standard deviation of the mean from the 256 independent Monte Carlo runs.

The contributions due to the uncertainties in the potentials were evaluated by calculating *C*(*T*) with perturbed upper- and lower-bound versions of the potentials as described in Sec. 2. In order to avoid noise introduced by the PIMC convergence uncertainty, these calculations were performed with the semiclassical method described in Sec. 3.1 of Ref. [4], which was shown to be fairly accurate down to about 50 K. This approach to estimating uncertainty is adequate even at lower temperatures where the semiclassical values of *C*(*T*) are no longer very accurate, since the needed quantity is not *C*(*T*) itself but rather the difference between *C*(*T*) calculated from the upper perturbed potential and *C*(*T*) calculated from the lower perturbed potential.

Below 20 K, the semiclassical results deviate sufficiently from reality that we no longer trust them for uncertainty analysis. Instead, we observe that the uncertainty due to the potentials increases slowly and smoothly as the temperature is reduced, while the statistical uncertainty of the PIMC calculation increases more quickly. Because of these trends, the contribution of the potential uncertainty, which is our largest uncertainty component above 40 K, is roughly 60 % as large as the PIMC convergence component at 20 K. It is reasonable to assume that this trend continues, so that the uncertainty from the potentials will be less than 60 % of that from the PIMC convergence at lower temperatures. Therefore, we make the conservative estimate that the potential component of the uncertainty is 60 % of that from the PIMC convergence at all temperatures below 20 K.

The last column of Table 1 shows the resulting expanded uncertainties *U*(*C*) with coverage factor *k* = 2. For purposes of illustration, we summarize the uncertainty calculation for the point at 273.16 K. The standard uncertainty of the PIMC integration is 0.0069 cm^6^ · mol^−2^. The standard uncertainty due to the uncertainty of the two-body potential is 0.0042 cm^6^ · mol^−2^ (1/4 of the difference between *C*(*T*) calculated semiclassically with the upper- and lower-bound pair potentials, with the three-body potential of Ref. [11] used in each case). The standard uncertainty due to the three-body potential, computed analogously with perturbed three-body potentials, is 0.0311 cm^6^ · mol^−2^. These are combined in quadrature to yield a standard uncertainty *u*(*C*) = 0.0321 cm^6^ · mol^−2^, which when multiplied by two yields an expanded uncertainty *U*(*C*) of 0.064 cm^6^ · mol^−2^.

The uncertainty in the acoustic third virial coefficient, shown in Table 2, is computed analogously. In this case, the analysis is less rigorous because the perturbed potentials do not necessarily define upper and lower bounds for the temperature derivatives of *C* that contribute to *γ*_a_. However, at all but the highest temperatures (above 2000 K), the uncertainty in *γ*_a_ is dominated by the convergence uncertainty in the PIMC calculations, so the expanded uncertainties shown in Table 2 should be good estimates.

### 4.4 Correlation for Results

We correlated the results for *C*(*T*) in Table 1 as a function of temperature:

(22) $$\frac{C}{1{\text{cm}}^{6}\cdot {\text{mol}}^{-2}}=\sum_{i=1}^{6}a_{i}{\left(T^{*}\right)}^{b_{i}},$$

where T* = *T*/(100 K) and the parameters *a_i_* and *b_i_* are given in Table 3. Equation (22) reproduces the values in Table 1 within tolerances smaller than their expanded uncertainties *U*(*C*); the fit is much closer than *U*(*C*) at high temperatures where the uncertainty is dominated by the Type B contribution from the uncertainty of the potentials. It covers the entire range from 2.6 K to 10 000 K, but should not be extrapolated outside this range. It may be differentiated to obtain d*C*/d*T* and d^2^*C*/d*T* ^2^, which can be used for calculation of acoustic virial coefficients as discussed in Sec. 4.2.

It is also convenient to have a continuous function for the expanded uncertainty *U* (*C*). This will naturally be approximate, as there is significant noise in the uncertainties given in Table 1. *U*(*C*) can be represented reasonably well as a function of temperature over the entire range of Table 1 by

(23) $$\log_{10}\left[\frac{U\left(C\right)}{1{\text{cm}}^{6}\cdot {\text{mol}}^{-2}}\right]=3.12-3.76\tau +1.07\tau^{2}-0.104\tau^{3},$$

where *τ* = log_10_(*T*/K).

### 4.5 Comparison With Experiment for *C*(*T*)

Extensive comparisons with experimental *C*(*T*) data were given in Ref. [4], demonstrating that the uncertainties of calculated *C*(*T*) were much smaller than those obtained from experiment. Since our new values are within the expanded uncertainties of those calculated previously, we do not repeat all the comparisons because the figures would look nearly identical to those in Ref. [4]. Instead, we limit our comparisons to the important range near room temperature and to the low-temperature range that was not covered in Ref. [4].

**Fig. 1:**
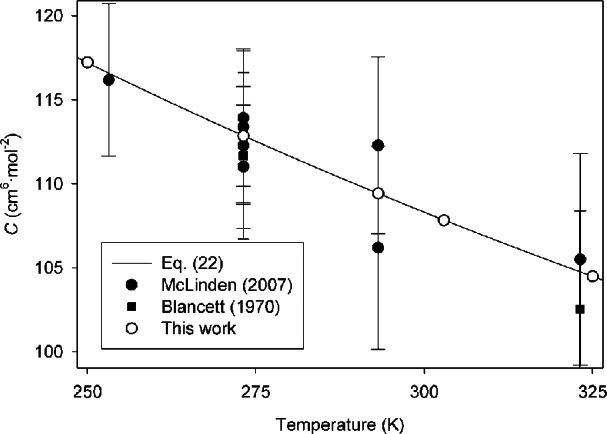
Comparison of *C*(*T*) for ^4^He calculated in this work with experimental values at near-ambient temperatures. Error bars on experimental points represent expanded uncertainties with coverage factor *k* = 2; uncertainties for this work (see Table 1) are not shown because the error bars would be smaller than the symbols.

In Fig. 1, our results are compared to those from the two most widely used experimental sources for *C*(*T*) [17,18] at temperatures from 250 K to 325 K. The error bars on the experimental points represent expanded uncertainties with coverage factor *k* = 2, while the expanded uncertainties on our calculated points (see Table 1) are smaller than the size of the symbols. Our calculations are fully consistent with the experimental data, but have smaller uncertainties by factors of approximately 50.

**Fig. 2:**
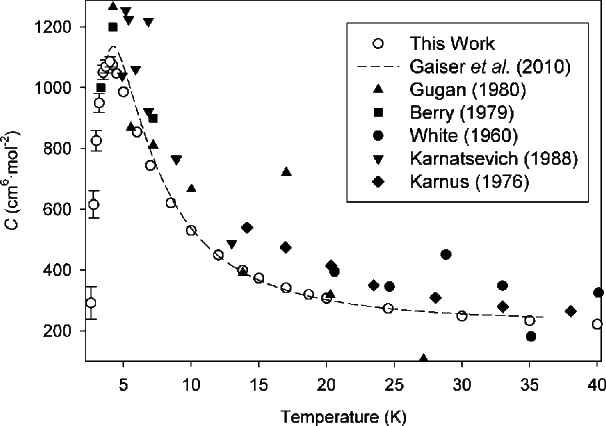
Comparison of *C*(*T*) for ^4^He calculated in this work with experimental values at low temperatures. Error bars on calculated points represent expanded uncertainties with coverage factor *k* = 2 (see Table 1), and are not shown when they would be smaller than the symbol size. Uncertainties for experimental points are not shown for clarity (see text).

In Fig. 2, we compare our calculated *C*(*T*) with the available experimental data below 40 K [19–24]. Error bars drawn on the points from this work represent expanded uncertainties *U*(*C*) from Table 1; they are not drawn above 5 K because they would be smaller than the size of the symbols. For clarity, we do not draw error bars for the experimental points; in some sources [19,20] these were not reported and in the others they were usually quite large (on the order of hundreds of cm^6^ · mol^−2^), often extending off the scale of Fig. 2. Gaiser et al. [24] described their data obtained by dielectric-constant gas thermometry from 3.7 K to 36 K with a smooth function for *C*(*T*), which we show as a dashed line on Fig. 2. From a figure in Ref. [24], it appears that their expanded (*k* = 2) uncertainties would be on the order of 20 cm^6^ · mol^−2^ over most of this range, becoming somewhat larger at the lowest temperatures.

**Fig. 3:**
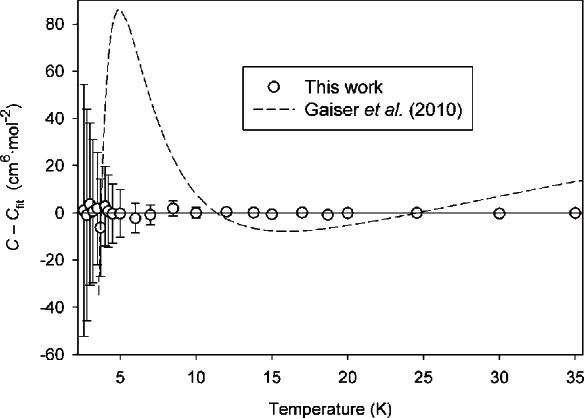
Deviation of PIMC results and of low-temperature *C*(*T*) data of Gaiser et al. [24] from fitted *C*(*T*) as given by Eq. (22).

Our results are generally consistent with the older experimental sources [19–23] within their scatter and uncertainties. We are for the most part in good agreement with the recent results of Gaiser et al. [24], with moderate disagreement at the low end of their temperature range. To examine this more closely, in Fig. 3 we plot the difference between the function of Gaiser et al. and our results as correlated by Eq. (22); Fig. 3 also shows our calculated PIMC points to demonstrate that Eq. (22) reproduces our results within their uncertainties. Our results agree with Gaiser et al. within mutual expanded uncertainties except between approximately 4 K and 8 K. It is possible that the functional form assumed for *C*(*T*) by Gaiser et al. [24] is not the right shape to represent this system.

### 4.6 Comparison With Experiment for *γ*_a_

**Fig. 4:**
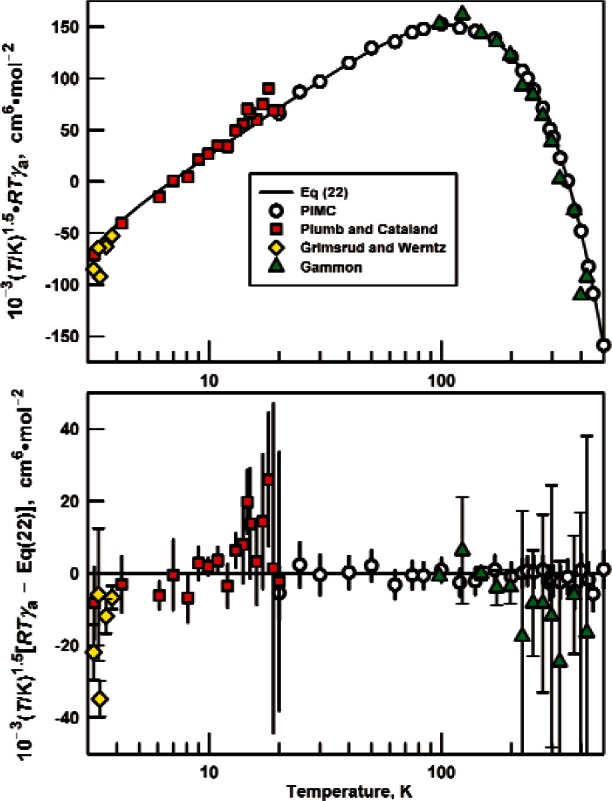
Comparison with experimental results of third acoustic virial coefficients *γ*_a_ for ^4^He calculated in this work both directly with path-integral Monte Carlo (PIMC), and with *γ*_a_ calculated with the use of derivatives of the *C*(*T*) correlation Eq. (22).

In this section, we compare our results for *RTγ*_a_ with measurements spanning the temperature range 3 K to 423 K. In this range, *RTγ*_a_ has a strong temperature dependence; it increases from approximately −13 000 cm^6^ · mol^−2^ at 3 K to approximately 900 cm^6^ · mol^−2^ near 14 K and then decreases to −9 cm^6^ · mol^−2^ at 423 K. Throughout most of this range, the uncertainty of our PIMC values of *RTγ*_a_ is on the order of 0.5 % to 3 %. Because of the wide range and precision required, we do not compare our results with measurements on a conventional graph. Instead, we have plotted the quantity 10^−3^(*T*/K)^1.5^ × *RTγ*_a_, where the exponent 1.5 was chosen so that the range of the product 10^−3^(*T*/K)^1.5^ × *RTγ*_a_ in the interval from 3 K to 423 K is much smaller than the range of *RTγ*_a_ (see Fig. 4).

We examined the speed-of-sound data for ^4^He published in archival journals and found three publications from which we could determine accurate values of *γ*_a_ [25–27]. Remarkably, the most recent of these publications is 35 years old. Thus, these studies did not benefit from the dramatic reduction in the uncertainty of speed-of-sound measurements that acoustic thermometry has achieved during the past 20 years [3, 28–30].

Gammon [25] measured the speed of sound of ^4^He on 14 isotherms spanning the temperature range 98 K to 423 K at intervals of 25 K and spanning the pressure range 10 atm to 150 atm at intervals of 10 atm (1 atm = 0.101 325 MPa). Gammon correlated his data using a classical model He-He pair potential. From his correlation, he identified the isotherm at 348 K and 10 other isolated measurements as outliers. We ignored Gammon’s isolated outliers and fit the remaining data on each isotherm, including 348 K, with the function

(24) $$Z_{a}-\frac{\beta_{a}p}{RT_{\text{lab}}}=\frac{T}{T_{\text{lab}}}+\frac{\gamma_{a}p^{2}}{RT_{\text{lab}}}+\frac{\delta_{a}p^{3}}{RT_{\text{lab}}}+\frac{\varepsilon_{a}p^{4}}{RT_{\text{lab}}}.$$

In Eq. (24), we define *Z*_a_ ≡ *Mu*^2^/(*γ*_0_*RT*_lab_) as the square of the speed of sound divided by its ideal-gas value, and we calculated it from Gammon’s tabulated values of *u*^2^. The values of *Z*_a_ have the narrow range 1 to 1.38; therefore, we weighted every data point on each isotherm equally. We took *β*_a_ from Cencek et al. [9], and we fitted the parameters *T*/*T*_lab_, *γ*_a_/(*RT*_lab_), and, when they are statistically unequal to zero, *δ*_a_/(*RT*_lab_) and *ε*_a_/(*RT*_lab_). [Here, *T* is the thermodynamic temperature of each isotherm and *T*_lab_ is the temperature reported by Gammon after adjustment for the changes in the internationally accepted temperature scale. The parameter *T*/*T*_lab_ also accounts for possible changes in *M*/*γ*_0_ that would occur if there were impurities in the helium.] Seven of Gammon’s 14 isotherms (98 K, 148 K, 173 K, 198 K, 248 K, 273 K, and 373 K) were very well behaved; that is, the deviations from a fit with an *appropriate* number of terms had no obvious pressure dependence. The standard deviation of *Z*_a_ − *β*_a_*p*/(*RT*_lab_) from the fits, averaged over these isotherms, was 0.000 014. For the remaining isotherms, the deviations from the fits are neither random nor have single, outlying points. Therefore, we are unable to rigorously estimate the uncertainties of *γ*_a_/*RT*. [We confirmed Gammon’s identification of the 348 K isotherm as anomalous because a satisfactory fit required the term *δ*_a_*p*^3^/(*RT*_lab_) even though the adjacent isotherms (323 K and 373 K) did not.] For the 13 isotherms (excluding 348 K), we estimated the standard uncertainty *u*(*γ*_a_/*RT*) by multiplying the result of the fitting routine by (*χ* ^2^/*N*)^1/2^, where *N* is the number of degrees of freedom and *χ* ^2^ is the sum of the squares of the deviations of the data from the function fitted to it. All of the tabulated (Table 4) uncertainties reflect multiplication of *u*(*RTγ*_a_) by an additional factor of two to approximate a 95 % confidence limit.

The values of *RTγ*_a_ and their expanded uncertainties *U*(*RTγ*_a_) resulting from fitting Gammon’s data are displayed in Fig. 4 and Table 4. Our calculations and Gammon's data agree within combined uncertainties, even though Gammon’s values of *RTγ*_a_ are more negative than our calculated values near the upper end of his temperature range. The uncertainties from fitting acoustic data will be underestimated if they do not account for the bias introduced by truncating the virial expansion. We crudely estimate the effect of truncation by comparing the 2nd and 4th columns in Table 4. For Gammon’s isotherms at 98 K, 123 K, 148 K, and 173 K, Table 4 compares *RTγ*_a_ obtained with and without the term *ε*_a_*p*^4^/(*RT*_lab_) in Eq. (24). The two values of *RTγ*_a_ agree within combined uncertainties. Table 4 also compares values of *RTγ*_a_ obtained with and without the term *δ*_a_*p*^3^/(*RT*_lab_) on the isotherms 198 K through 298 K. Except at 298 K, the two values of *RTγ*_a_ on each isotherm are mutually consistent. Above 298 K, *δ*_a_ was zero, within its uncertainty; however, the values of *RTγ*_a_ could be influenced by contributions from *δ*_a_. We verified that the uncertainties of the values of a *β*_a_ from Cencek et al. [9] did not contribute significantly to the uncertainty of *γ*_a_.

Plumb and Cataland [27] measured the speed of sound in ^4^He on 21 isotherms from 2.3 K to 20 K for the purpose of determining the thermodynamic temperature *T*. As is often done in acoustic thermometry, Plumb and Cataland deliberately restricted their data to low densities; therefore, they were unable to determine meaningful values of *γ*_a_. Because we have *β*_a_ from Cencek et al. [9], we were able to determine values of *γ*_a_ on all of the isotherms except those at 5 K, 2.8 K, and 2.3 K. For each isotherm, the values of *δ*_a_ and *ε*_a_ in Eq. (24) were set equal to zero, and the data were weighted equally. The results are displayed on Fig. 4 and in Table 5.

Grimsrud and Werntz [26] measured the speed of sound in ^4^He on eight isotherms from 2.13 K to 3.816 K. They fitted their data by, in effect, adjusting *T*/*T*_lab_, *β*_a_, and *γ*_a_. When analyzing their data, we weighted each measurement using the uncertainties that they tabulated. Because we fixed values of *β*_a_ from Cencek et al. [9], we were able to determine values of *γ*_a_ with roughly 1/5 the uncertainty achieved by Grimsrud and Werntz. These values are also shown in Table 5 and on Fig. 4. The values of *RTγ*_a_ from Grimsrud and Werntz are systematically more negative than our calculated values, particularly at the lowest temperatures where, for reasons discussed in the next section, the measurements may be more accurate than our calculations.

In the narrow region of overlap, the data of Grimsrud and Werntz [26] are consistent with the data of Plumb and Cataland [27]. As the temperature decreases, both sets of data tend towards values of *RTγ*_a_ smaller than those derived from our Eq. (22).

Additional values for *γ*_a_ derived from acoustic experiments between 2.3 K and 34 K were reported in a conference proceeding by Plumb [31]. Unfortunately, the actual measured data were never reported, so we were not able to apply new high-accuracy values of *β*_a_ [9] to obtain values of *γ*_a_ consistent with the best current knowledge, as we did for Refs. [25–27]. We therefore do not show the data from Ref. [31] in Fig. 4, but we note that the reported values of *γ*_a_ are generally consistent with our calculated values within the reported uncertainties of Ref. [31]. Our calculated values are also consistent within mutual uncertainties with a recent experimental result for *γ*_a_ at 273.16 K by Gavioso et al. [32].

### 4.7 Results for ^3^He

While the primary focus of this work was on the common isotope ^4^He, the same methods can be used for ^3^He, which is of interest for cryogenic temperature metrology. Table 6 presents values of *C*(*T*) for ^3^He, along with their expanded uncertainties. More extensive discussion of the ^3^He calculations at low temperatures, along with comparison with the limited experimental data, is given in Ref. [12]. We note that, in addition to the data sources below 10 K examined in Ref. [12], values of *C*(*T*) for ^3^He between 14 K and 60 K were reported by Karnus [20]; these seem to be systematically high below about 30 K, similar to the data for ^4^He from the same study shown in Fig. 2.

For the same reason discussed for ^4^He in Sec. 4.1, the values in Table 6 differ slightly from those reported in Ref. [12], and the new values in Table 6 should be preferred.

### 4.8 Accuracy of Semiclassical Calculations

In Ref. [4], we assessed the accuracy of a first-order semiclassical calculation of *C*(*T*), concluding that the semiclassical results were adequate (in the sense of reproducing the fully quantum *C*(*T*) from PIMC calculations within their expanded uncertainties at the *k* = 2 level) at temperatures above about 120 K. That conclusion can be reassessed in light of the reduced uncertainties achieved in this work. The semiclassical *C*(*T*) deviates from our new PIMC results by more than the expanded uncertainty of our new results at temperatures below about 280 K. For example, at 273.16 K, the semiclassical calculation yields 112.939 cm^6^ · mol^−2^, which exceeds the PIMC value by slightly more than the expanded uncertainty given in Table 1.

## 5. Discussion

The availability of new, state-of-the-art pair and three-body potentials has allowed us to calculate *C*(*T*) for helium with uncertainties approximately one-fourth that of our previous work [4]. In addition, we have extended the temperature range of our results, which previously had a lower bound of 24.5661 K, to 2.6 K. We also calculated *C*(*T*) for the ^3^He isotope. The incorporation of exchange effects (non-Boltzmann statistics) was necessary to achieve accurate results for both isotopes below about 7 K.

Within the temperature range covered in our previous work [4], our new results given in Table 1 are consistent with our previous results. The present *C*(*T*) are somewhat higher than those calculated previously, typically by an amount near one-half of the expanded (*k* = 2) uncertainties of the results in Ref. [4]. This change is primarily due to the more accurate three-body potential used here [11], and is consistent with a few preliminary calculations using the potential of Ref. [11] that were reported in Ref. [4].

We extended the PIMC method to calculate the third acoustic virial coefficient *γ*_a_; our results are consistent with values of *γ*_a_ obtained from experimental acoustic data, and have smaller uncertainties above 18 K. However, the statistical uncertainty in the PIMC calculation of *γ*_a_ becomes quite large at lower temperatures. We believe more reliable values are obtained by differentiating Eq. (22) to obtain the temperature derivatives of *C* and then using those values in Eq. (11). Values derived in that way are consistent with experimental results for *γ*_a_ in the entire range of our *C*(*T*) correlation, with the possible exception of the lowest temperatures (below 4 K), where the experimental data lie slightly below our results. One might expect this approach to be less reliable at the low end of the temperature range of Eq. (22), where the uncertainty in the points to which the *C*(*T*) function was fitted is larger and d*C*/d*T* and d^2^*C*/d*T*^2^ derived from Eq. (22) would have relatively large uncertainties.

At low temperatures, the uncertainty of the present results for *C*(*T*) is dominated by the statistical uncertainty of the PIMC integration. This could, of course, be improved somewhat simply by applying more computing resources. Above about 35 K, the uncertainty from the three-body potential becomes the largest contribution. Therefore, for metrology near room temperature, further improvement in the three-body potential would be desirable; a reduction in by a factor of two in the uncertainty of the three-body potential would produce a reduction by almost that factor in the uncertainty of *C*(*T*) at room temperature.

The present results could also be extended to lower temperatures at the expense of more computer time. This could have some application in primary thermometry at these temperatures.

One could perform similar calculations for the “cross” third virial coefficients that characterize isotopic mixtures; these would be *C*_334_(*T*), representing interactions among two ^3^He atoms and one ^4^He atom, and the similarly defined *C*_344_(*T*). Because the natural abundance of ^3^He is tiny, contributions from these coefficients would be insignificant for experiments with naturally occurring helium. We are not aware of any situation in metrology where these mixture coefficients would be useful, but, if needed, the extension of the methods used here to the mixture coefficients would be straightforward.

Another quantity of interest is the fourth virial coefficient *D*(*T*), whose calculation would be a straightforward extension of our methods. With the reduction in the uncertainty of *C*(*T*) achieved in the present work, *D*(*T*) will become the largest uncertainty in some situations in metrology [7]. In principle, calculating *D*(*T*) requires not only pair and three-body potentials but also the nonadditive four-body potential. Such a potential would be difficult to develop, but the relatively small size of the three-body effects in helium suggests that one might be able to assume the nonadditive four-body effects were negligible. It should be possible to test that assumption by performing a few high-level *ab initio* calculations for simple assemblies of four helium atoms (such as tetrahedrons or squares). The calculation for *D*(*T*) would require major computing resources because of the increased dimensionality of the integral, but such a calculation may at least be feasible near room temperature where the number of beads in the ring polymers in the PIMC procedure would be relatively small.

## Figures and Tables

**Table 1:** Third virial coefficients *C*(*T*) for ^4^He calculated in this work and our estimates (see Sec. 4.3) of their expanded (*k* = 2) uncertainties U(C)

*T* (K)	*C* (cm^6^ · mol^−2^)	*U*(*C*) (cm^6^ · mol^−2^)
2.6	292.	53.
2.8	616.	45.
3	826.	35.
3.2	950.	30.
3.5	1050.	24.
3.7	1070.	21.
4.0	1086.	17.
4.2	1075.	15.
4.5	1047.	13.
5	987.	10.
6	854.8	6.2
7	744.5	4.2
8.5	620.7	3.2
10	530.1	2.3
12	449.9	1.5
13.8033	399.8	1.1
15	373.8	1.0
17	341.91	0.82
18.689	319.86	0.70
20	307.24	0.64
24.5561	273.84	0.47
30	248.75	0.37
35	233.56	0.31
40	222.08	0.27
50	205.75	0.21
63.15	190.87	0.17
75	180.81	0.15
83.806	174.59	0.14
100	164.84	0.12
120	155.06	0.10
140	146.846	0.092
170	136.791	0.082
200	128.427	0.074
223.152	122.916	0.070
235	120.266	0.068
250	117.223	0.067
273.16	112.847	0.064
293.15	109.426	0.062
302.915	107.822	0.062
325	104.493	0.060
350	100.997	0.058
375	97.793	0.057
400	94.837	0.056
429.75	91.598	0.055
450	89.552	0.054
500	84.934	0.053
550	80.885	0.051
600	77.285	0.050
650	74.039	0.050
700	71.104	0.049
750	68.427	0.048
800	65.994	0.048
900	61.661	0.047
1000	57.949	0.047
1200	51.857	0.046
1400	47.039	0.045
1600	43.114	0.045
1800	39.842	0.044
2000	37.063	0.044
2500	31.615	0.043
3000	27.610	0.042
4000	22.039	0.041
5000	18.317	0.039
7500	12.765	0.036
10000	9.664	0.034

**Table 2:** Third acoustic virial coefficients expressed as *RTγ*_a_ for ^4^He calculated by PIMC and our estimates of their expanded (*k* = 2) uncertainties *U*(*C*). Also shown are values calculated from the *C*(*T*) correlation Eq. (22)

*T* (K)	*RTγ*_a_ (PIMC) (cm^6^ · mol^−2^)	*U*(*RTγ*_a_) (cm^6^ · mol^−2^)	*RTγ*_a_ (Eq. 22) (cm^6^ · mol^−2^)
3	–	–	−13335.
4	–	–	−5250.
5	–	–	−2124.
6	–	–	−681.
7	–	–	57.
8	–	–	458.
9	–	–	682.
10	–	–	807
12	–	–	906.
14	–	–	914.
16	–	–	885.
18	–	–	843.
20	736.	83.	796.
24.5561	715.	51.	695.
30	590.	34.	592.
40	455.	18.	454.
50	366.	12.	360.
63.15	269.9	7.8	276.4
75	222.9	6.0	223.9
83.806	192.9	4.8	193.9
100	152.7	3.3	151.9
120	113.4	2.8	115.4
140	88.0	2.2	89.3
170	62.4	1.8	61.9
200	42.8	1.4	43.1
223.152	32.3	1.2	32.3
235	27.8	1.1	27.6
250	22.5	1.1	22.4
273.16	15.82	0.88	15.61
293.15	10.18	0.93	10.71
302.915	8.18	0.79	8.59
325	3.90	0.75	4.32
350	0.07	0.73	0.25
375	−3.90	0.65	−3.19
400	−6.03	0.57	−6.12
429.75	−9.27	0.52	−9.06
450	−11.39	0.50	−10.79
500	−14.19	0.46	−14.31
550	−17.65	0.40	−17.02
600	−19.31	0.43	−19.12
650	−20.90	0.36	−20.79
700	−21.93	0.34	−22.11
750	−22.93	0.32	−23.17
800	−23.98	0.32	−24.02
900	−25.27	0.26	−25.26
1000	−26.28	0.25	−26.07
1200	−26.99	0.23	−26.87
1400	−27.13	0.21	−27.07
1600	−27.13	0.19	−26.96
1800	−26.77	0.18	−26.68
2000	−26.40	0.17	−26.30
2500	−25.24	0.14	−25.19
5000	−20.27	0.11	−20.25
10000	−14.58	0.09	−14.72

**Table 3:** Coefficients for Eq. (22) for the third virial coefficient of helium

*i*	*a_i_*	*b_i_*
1	177.98	−0.15
2	−494.87	−0.25
3	849.84	−0.50
4	−1003.30	−0.95
5	635.18	−1.07
6	−0.035 012	−3.15

**Table 4:** Third acoustic virial coefficient for ^4^He and its expanded (*k* = 2) uncertainty from the data of Gammon [25], excluding the isotherm at 348 K. Asterisks indicate fits including additional terms, as discussed in the text

*T* K	*RTγ*_a_ cm^6^·mol^−2^	*U*(*RTγ*_a_) cm^6^·mol^−2^	(*RTγ*_a_)* cm^6^·mol^−2^	[*U*(*RTγ*_a_)]* cm^6^·mol^−2^
98.15	153.2^a^	1.0	155.1^b^	2.5
123.16	106.0^a^,^c^	6.3	115.3^b^	10.5
148.16	77.5^a^	1.0	80.7^b^	2.5
173.16	57.8^a^	2.1	57.9^b^	8.0
198.16	32.4	4.1	42.8^a^	1.6
223.16	22.4	2.3	27.0^a^	10.2
248.16	17.1	0.9	20.9^a^	3.7
273.15	8.2	1.3	13.8^a^	5.3
298.15	7.3	6.9	21.2^a^	27.4
323.13	0.4	4.7		
373.13	−3.8	11.9		
398.15	−13.5	40.5		
423.15	−10.4	26.0		

a Fit included the term *δ*_a_*p*^3^ / (*RT*_lab_).

b Fit included the terms *δ*_a_*p*^3^ / (*RT*_lab_) and *ε*_a_*p*^4^ / (*RT*_lab_).

c Not recommended.

**Table 5:** Third acoustic virial coefficient for ^4^He and its expanded (*k* = 2) uncertainty from the data of Grimsrud and Werntz [26] and from Plumb and Cataland [27]

*T* K	*RTγ*_a_ cm^6^·mol^−2^	*U*(*RTγ*_a_) cm^6^·mol^−2^
Grimsrud and Werntz [26]		
3.816	−7100	440
3.595	−9300	700
3.379	−14800	790
3.337	−10600	3000
3.182	−15000	1400
2.978	−16400	1300
2.671	−19500	4300
2.13	−46000	7500
Plumb and Cataland [27]		
20	770	400
18.9	840	560
18	1180	240
17	1070	270
16	940	190
15	1140	260
14.6	1260	160
14	1070	210
13	1050	100
12	820	140
10.9	970	110
9.9	854	75
9	780	170
8.1	190	300
7	30	530
6.1	−990	260
4.2	−4740	910
3.2	−12400	1800
5	1400	5100

**Table 6:** Third virial coefficients *C*(*T*) for ^3^He calculated in this work and our estimates (see Sec. 4.3) of their expanded (*k* = 2) uncertainties *U*(*C*)

*T* K	*C* cm^6^·mol^−2^	*U*(*C*) cm^6^·mol^−2^
2.6	1343.	62.
2.8	1436.	46.
3	1464.	41.
3.2	1462.	36.
3.5	1418.	29.
3.7	1366.	27.
4.0	1298.	22.
4.2	1257.	19.
4.5	1175	16.
5	1071.	12.
6	895.0	9.1
7	775.6	6.6
8.5	644.9	4.4
10	553.7	3.4
12	475.3	2.4
13.8033	426.5	1.7
15	401.8	1.6
17	367.6	1.3
18.689	346.9	1.0
20	333.53	0.87
24.5561	296.76	0.67
30	268.91	0.52
35	251.22	0.41
50	218.56	0.26
100	170.63	0.13
150	146.71	0.10
200	130.762	0.081
273.16	114.365	0.068
300	109.627	0.065
400	95.707	0.058
500	85.563	0.054
750	68.758	0.049
1000	58.168	0.047
1500	45.083	0.045
2000	37.130	0.044
