# Erratum: Improved First-Principles Calculation of the Third Virial Coeffcient of Helium

**DOI:** 10.6028/jres.125.019

**Published:** 2020-06-06

**Authors:** Giovanni Garberoglio, Michael R. Moldover, Allan H. Harvey

**Affiliations:** 1 European Centre for Theoretical Studies in Nuclear Physics and Related Areas (FBK-ECT*) and Trento Institute for Fundamental Physics and Applications (TIFPA-INFN), Trento, I-38123 Italy; 2 Sensor Science Division, National Institute of Standards and Technology, Gaithersburg, MD 20899 USA; 3 Applied Chemicals and Materials Division, National Institute of Standards and Technology, Boulder, CO 80305 USA

**Keywords:** helium, virial coefficients

## Erratum

The calculations of $C\left(T\right)$ for ^3^He in the original paper  [1] were in error at the lowest temperatures due to an incorrect accounting for the quantum statistics of fermions, as explained in Ref. [2]. A corrected version of Table 6 is given below. The calculations for ^4^He are not affected by this error.

**Table 6:** Third virial coefficients *C*(*T*) for ^3^He calculated in this work and our estimates (see Sec. 4.3) of their expanded (*k* = 2) uncertainties *U*(*C*).

*T* K	*C* cm^6^·mol^−2^	*U*(*C*) cm^6^·mol^−2^
2.6	1659	55
2.8	1648	42
3	1605	36
3.2	1560	32
3.5	1473	26
3.7	1403	24
4	1321	20
4.2	1274	17
4.5	1185	15
5	1075	11
6	896.2	8.1
7	775.9	5.9
8.5	644.9	4.0
10	553.7	3.0
12	475.3	2.4
13.8033	426.5	1.7
15	401.8	1.6
17	367.6	1.3
18.689	346.9	1.0
20	333.53	0.87
24.5561	296.76	0.67
30	268.91	0.52
35	251.22	0.41
50	218.56	0.26
100	170.63	0.13
150	146.71	0.10
200	130.762	0.081
273.16	114.365	0.068
300	109.627	0.065
400	95.707	0.058
500	85.563	0.054
750	68.758	0.049
1000	58.168	0.047
1500	45.083	0.045
2000	37.130	0.044
